# MoSGrid: efficient data management and a standardized data exchange format for molecular simulations in a grid environment

**DOI:** 10.1186/1758-2946-4-S1-P21

**Published:** 2012-05-01

**Authors:** Georg Birkenheuer, Dirk Blunk, Sebastian Breuers, André Brinkmann, Ines dos Santos Vieira, Gregor Fels, Sandra Gesing, Richard Grunzke, Sonja Herres-Pawlis, Oliver Kohlbacher, Jens Krüger, Ulrich Lang, Lars Packschies, Ralph Müller-Pfefferkorn, Patrick Schäfer, Thomas Steinke, Klaus-Dieter Warzecha, Martin Wewior

**Affiliations:** 1 Universität Paderborn, Paderborn 33098, Germany; 2 Universität zu Köln, Köln 50923, Germany; 3 Fakultät Chemie, Technische Universität Dortmund, Dortmund 44221, Germany; 4 Eberhard-Karls-Universität Tübingen, Tübingen 72074, Germany; 5 Technische Universität Dresden, Dresden 01187, Germany; 6 Konrad-Zuse-Zentrum für Informationstechnik, Berlin 14195, Germany

## 

The MoSGrid (Molecular Simulation Grid) project is currently establishing a platform that aims to be used by both experienced and inexperienced researchers to submit molecular simulation calculations, monitor their progress, and retrieve the results. It provides a web-based portal to easily set up, run, and evaluate molecular simulations carried out on D-Grid resources. The range of applications available encompasses quantum chemistry, molecular dynamics, and protein-ligand docking codes.

In addition, data repositories were developed, which contain the results of calculations as well as “recipes” or workflows. These can be used, improved, and distributed by the users. A distributed high-throughput file system allows efficient access to large amounts of data in the repositories. For storing both the input and output of the calculations, we have developed MSML (Molecular Simulation Markup Language), a CML derivative (Chemical Markup Language). MSML has been designed to store structural information on small as well as large molecules and results from various molecular simulation tools and docking tools. It ensures interoperability of different tools through a consistent data representation.

At http://www.mosgrid.de the new platform is already available to the scientific community in a beta test phase. Currently, portlets for generic workflows, Gaussian, and Gromacs applications are publicly accessible [1,2].
